# Development of the CK‐MB‐1 trastuzumab‐resistant HER2‐positive breast cancer cell line and xenograft animal models

**DOI:** 10.1002/cam4.3824

**Published:** 2021-03-05

**Authors:** Wei‐Pang Chung, Wei‐Lun Huang, Wei‐An Liao, Wan‐Ling Huang, You‐Yu Liu, Wu‐Chou Su

**Affiliations:** ^1^ Institute of Clinical Medicine College of Medicine National Cheng Kung University Tainan Taiwan; ^2^ Department of Oncology National Cheng Kung University Hospital College of Medicine National Cheng Kung University Tainan Taiwan; ^3^ Center of Applied Nanomedicine National Cheng Kung University Tainan Taiwan; ^4^ Department of Pathology National Cheng Kung University Hospital College of Medicine National Cheng Kung University Tainan Taiwan

**Keywords:** animal models, breast cancer, cell lines, trastuzumab, xenograft

## Abstract

**Background:**

Patients with human epidermal growth factor receptor 2 (HER2)‐positive breast cancer who fail to respond to anti‐HER2 treatments have poor prognoses. Most trastuzumab‐resistant breast cancer cell lines available from biobanks feature either *phosphoinositide‐3‐kinase*, *catalytic*, *alpha* (*PIK3CA*) mutation or the loss of phosphatase and tensin homolog (PTEN). However, *PIK3CA* mutations and/or PTEN loss do not account for most trastuzumab‐resistant tumors in humans.

**Methods:**

Breast cancer cells were collected from one patient's malignant ascites. These cells were cultured and maintained to develop a stable cell line, which we named CK‐MB‐1. We used western blotting to evaluate protein expression. The *PIK3CA* status of CK‐MB‐1 cells was analyzed using Sanger sequencing and validated using next‐generation sequencing. In vivo, CK‐MB‐1 xenograft tumor models were developed in zebrafish and immunodeficient mice.

**Results:**

CK‐MB‐1 cells maintained the major characteristics of the parental tumor including HER2 positivity and estrogen receptor negativity. The HER2 gene amplification of CK‐MB‐1 cells was detected by fluorescence in situ hybridization. The integrity of PTEN was confirmed by its positive protein expression and the absence of gene mutations. No common *PIK3CA* mutation was detected. Compared with the findings in two other HER2‐positive trastuzumab‐resistant cell lines, CK‐MB‐1 cells exhibited greater resistance to trastuzumab, chemotherapeutics, and small‐molecule drugs. Trastuzumab resistance in CK‐MB‐1 cells was confirmed in vivo using the NOD SCID mouse model.

**Conclusions:**

CK‐MB‐1 cells represent a stable HER2‐positive trastuzumab‐resistant breast cancer cell line. The resistance of CK‐MB‐1 cells does not originate from the PTEN or phosphoinositide 3‐kinase signaling pathway, which can provide an alternative approach for potential drugs.

## Graphical abstract

Compared with the findings in two other HER2‐positive cell lines, CK‐MB‐1 cells exhibited greater resistance to trastuzumab, chemotherapeutics, and small‐molecule drugs, and the resistance of the cells to trastuzumab was confirmed in zebrafish and mouse models. Because the resistance of the cell line to trastuzumab is not associated with alterations in PI3K or PTEN signaling, these cells should be useful for developing novel treatments for drug‐resistant breast cancer.

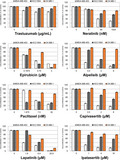

## 1 INTRODUCTION

As the most common type of malignancy among women globally, approximately 20% of patients with breast cancer exhibit gene amplification and/or overexpression of human epidermal growth factor receptor 2 (HER2).^1^ This breast cancer subtype once carried the poorest prognosis. Currently, the outcomes of these patients have been dramatically improved by the development of anti‐HER2 monoclonal antibodies, tyrosine kinase inhibitors, and antibody‐drug conjugates.^2^, ^3^, ^4^, ^5^ The next step in improving the prognosis of HER2‐positive breast cancer depends on proper treatment after the failure of anti‐HER2 therapies. Several hypotheses have been proposed to address the mechanism of resistance, including mutation of *phosphoinositide‐3‐kinase*, *catalytic*, *alpha* (*PIK3CA*).^6^ The Food and Drug Administration of the United States has approved alpelisib, an alpha isoform‐specific phosphoinositide 3‐kinase (PI3K) inhibitor, for the treatment of hormone receptor‐positive/HER2‐negative metastatic breast cancer based on the successful results of the SOLAR‐1 study.^7^ Patients with HER2‐positive breast cancer bearing *PIK3CA* mutations might benefit from this category of drug. However, patients with *PIK3CA* mutations and/or the loss of phosphatase and tensin homolog (PTEN) account for less than 40% of the trastuzumab‐resistant HER2‐positive population.^8^ The treatment strategy for this group after the failure of trastuzumab remains unclear.

A proper cell line with established animal models is crucial for testing the clinical response to anticancer drugs.^9^ From the development of the antibody‐drug conjugates T‐DM1 and DS‐8201a, we know that trastuzumab‐resistant cells were selected from resistant clones of the BT‐474 cell line or *PIK3CA*‐mutated and/or PTEN‐deleted cell lines.^10^, ^11^ There is no treatment information for these two drugs in parental HER2‐positive, *PIK3CA*/*PTEN*–wild‐type, trastuzumab‐resistant cells. Indeed, trastuzumab‐resistant breast cancer models can be obtained from transgenic mice or patient‐derived xenografts,^11^, ^12^ and certain types of tumors can then be further identified. However, the expense and facility requirements cannot be met by every research group. Therefore, isolating and characterizing a parental HER2‐positive cell line without altering PI3K or PTEN signaling and establishing associated tumor xenograft models should be helpful in the development of anticancer drugs in the future.

In this study, we reported the characterization and application of the CK‐MB‐1 cell line, which was harvested from a female patient with breast cancer who received several lines of anti‐HER2 therapies but still developed progressive disease. CK‐MB‐1 cells reflect the multidrug‐resistant properties of the parental tumor, and they can be used to establish xenograft tumors in animal models. We hope that the establishment of this cell line will be useful for testing drugs targeting trastuzumab‐resistant tumors.

## 2 METHODS

### 2.1 The patient and the harvest of breast cancer cells

Breast cancer cells were collected from a patient with breast cancer‐associated malignant ascites who was treated at National Cheng Kung University Hospital. The patient provided written informed consent for the use of her cells. The sample was verified to be positive for malignant cells via cytological analysis of the ascites. The ascites was collected and centrifuged immediately. The cancer cells were separated from malignant ascites‐associated lymphocytes via serial gradient centrifugation using Histopaque 1077 and Percoll (Merck KGaA, Darmstadt, Germany) as previously described.^13^, ^14^ The protocol for this study was approved by the institutional review board of National Cheng Kung University Hospital. The cancer cells were then maintained in RPMI‐1640 medium (Gibco by Life Technologies, Waltham, MA, USA) supplemented with 10% fetal bovine serum (FBS) (Gibco by Life Technologies). After more than 20 passages in cell culture dishes, the purity (the ratio of cancer cells in the cultured population) was near 100%, and the doubling time was stable. This cell line was named CK‐MB‐1 and then used for subsequent experimentation.

### 2.2 Cell lines, cell culture, and reagents

MCF7 and MDA‐MB‐231 cells were both obtained from the American Type Culture Collection (ATCC, Manassas, VA, USA) and maintained in Dulbecco's Modified Eagle's Medium (Gibco by Life Technologies) containing 10% FBS. BT‐474 cells were obtained from Bioresource Collection and Research Center (BCRC, Taiwan) and maintained in Hybri‐Care medium (ATCC) supplemented with 10% FBS. SK‐BR‐3 cells were obtained from ATCC and maintained in McCoy's 5A medium (Merck KGaA) supplemented with 10% FBS. HCC1569 and HCC1954 cells were both obtained from ATCC and maintained in RPMI‐1640 medium supplemented with 10% FBS. MDA‐MB‐453 cells were obtained from BCRC and maintained in Leibovitz's L‐15 medium (Merck KGaA) supplemented with 10% FBS. All cell lines were maintained at 37°C in an atmosphere of 5% carbon dioxide excluding MDA‐MB‐453 cells, which were maintained at 37°C without carbon dioxide supplementation. All cell lines have been authenticated with short tandem repeat profiling. Experiments were performed with mycoplasma‐free cells. Trastuzumab was purchased from the pharmacy at National Cheng Kung University Hospital and manufactured by Genentech (San Francisco, CA, USA). Lapatinib, neratinib, alpelisib, capivasertib, and ipatasertib were purchased from Selleck Chemicals (Houston, TX, USA) and prepared in DMSO. Epirubicin (manufactured by Pfizer, Bentley, Western Australia, Australia) and paclitaxel (manufactured by Sinphar Pharmaceutical, Yilan, Taiwan) were obtained from the pharmacy at National Cheng Kung University Hospital.

### 2.3 Western blotting

For cell lysis, the harvested samples were incubated on ice in whole cell extract lysis buffer for 30 minutes. Lysates were centrifuged at 12,000 rpm for 10 minutes, and the protein concentration was measured using the Bradford assay (Bio‐Rad, Hercules, CA, USA). For western blotting, 15–100 μg of lysates (depend on the target proteins assayed) were then boiled for 5 minutes with sample buffer before being separated on SDS‐polyacrylamide gels. Proteins were transferred to polyvinylidene difluoride membranes (Millipore, Billerica, MA, USA) and blocked with 5% nonfat milk/TBST buffer. The primary antibodies used were as follows: HER2, beta‐actin (Merck KGaA), ERα (Santa Cruz Biotechnology, Santa Cruz, CA, USA), progesterone receptor (PR) A/B, epidermal growth factor receptor (EGFR), and PTEN (Cell Signaling Technology, Beverly, MA, USA). Anti‐rabbit and anti‐mouse secondary antibodies were purchased from Jackson ImmunoResearch (West Grove, PA, USA).

### 2.4 HER2 fluorescence in situ hybridization

CK‐MB‐1 cells were collected by trypsinization, fixed with formalin, and embedded using paraffin for slide preparation. Formalin‐fixed paraffin‐embedded (FFPE) samples were then cut into 4‐µm sections and placed on slides. FFPE samples were then dehydrated by a xylene washing step followed by 100% ethanol. After drying at room temperature, slides were incubated with 0.2‐N hydrochloric acid followed by distilled water wash. Then, slides were incubated 8–10 minutes with VP2000 protease solution (Abbott, Abbott Park, IL, USA) then 5 minutes with pretreatment wash buffer. Dehydration was conducted by increasing ethanol concentration (70%, 85%, and 100%). After drying at room temperature, HER2 and chromosome enumeration probe 17 (CEP17) probes (PathVysion HER2 DNA Probe Kit II, Abbott) were hybridized in a wet chamber overnight at 37°C. Slides were then washed with two times saline‐sodium citrate buffer. Images were taken using fluorescence microscope (Axioskop 2, Zeiss, Oberkochen, Germany) after 4’,6‐diamidino‐2‐phenylindole counterstaining was conducted. Forty CK‐MB‐1 cells were picked for the analysis of HER2 copy number and HER2/CEP17 ratio. The interpretation of HER2 amplification was determined by the American Society of Clinical Oncology (ASCO)/College of American Pathologists (CAP) HER2 testing guideline in breast cancer.^15^

### 2.5 *PIK3CA* sequencing

We analyzed exons 9 and 20 of *PIK3CA* via PCR amplification of genomic DNA from CK‐MB‐1 cells and direct sequencing. The primers for *PIK3CA* were as follows: exon 9 forward, TTG CTT TTT CTG TAA ATC ATC T; exon 9 reverse, CTG CTT TAT TTA TTC CAA TAG G; exon 20 forward, CTC AAT GAT GCT TGG CTC TG; and exon 20 reverse, TGG AAT CCA GCG TGA GCT TTC. All sequencing was performed using an ABI 3500 Dx Genetic Analyzer.

### 2.6 Screening for major oncogenic alterations

The major oncogenic alterations in CK‐MB‐1 cells were analyzed via next‐generation sequencing (NGS) using Human Breast Cancer GeneRead DNAseq Targeted Panel V2 (Qiagen, Hilden, Germany) according to the manufacturer's instructions.^16^ The panel consists of PCR primers for the targeted enrichment of 2915 amplicons, which cover the coding regions of 44 genes commonly mutated in breast cancer, namely, *ACVR1B*, *CDH1*, *EXOC2*, *ITCH*, *NCOR1*, *PTEN*, *WEE1*, *AKT1*, *CDKN2A*, *EXT2*, *KMT2C*, *NEK2*, *PTGFR*, *ZBED4*, *ATM*, *EGFR*, *FBXO32*, *MAP2K4*, *PBRM1*, *RB1*, *BAP1*, *EP300*, *FGFR1*, *MAP3K1*, *PCGF2*, *RET*, *BRCA1*, *ERBB2*, *FGFR2*, *MDM2*, *PIK3CA*, *SEPT9*, *BRCA2*, *ERBB3*, *GATA3*, *MUC16*, *PIK3R1*, *TP53*, *CBFB*, *ESR1*, *IRAK4*, *MYC*, *PPM1L*, and *TRAF5*.

### 2.7 In vitro antiproliferation activity analysis

Cells were seeded at concentrations of 1.5 × 10^4^–3 × 10^4^ cells/200 μl/well in 96‐well plates for 24 hours and treated with the indicated agents for 72 hours. After treatment, the MTT proliferation assay was performed according to the manufacturer's instructions. Briefly, 20 µl of MTT reagent (5 mg/ml, Merck KGaA) were added into each well and incubated for 3 hours. The results were determined by measuring the absorbance at 490 nm. Experiments were repeated at least three times to confirm the reliability of the results.

### 2.8 Zebrafish (*Danio rerio*) heterotopic xenograft tumor models

Tg(fli1: EGFP) transgenic zebrafish (Taiwan Zebrafish Core Facility, National Health Research Institutes, Taiwan) were maintained in a breeding system as previously described.^17^ The zebrafish embryos were incubated in Petri dishes with filtered tap water at 28℃. Two days after fertilization, chorions of embryos were removed manually. Embryos were anesthetized with 0.016% tricaine (Merck KGaA) before they were microinjected with CK‐MB‐1 cells (500 cells/embryo) prepared in serum‐free medium at a density of 5 × 10^6^ cells/ml (*n* = four per group). To visualize the tumor cells injected in the embryos, tumor cell membranes were stained with Vybrant cell‐labeling solution (v22888, Thermo Fisher Scientific, Waltham, MA, USA) for 15 minutes. Cells were then redissolved in PBS at a density of 5 × 10^6^ cells/23 μl, and then phenol red (0.05%) was added. Nanoject II Auto Nanoliter Injectors (Daigger Scientific, Vernon Hills, IL, USA) were used to inject suspended CK‐MB‐1 cells (2.3 nl with 500 tumor cells/embryo) into the yolk sacs of Tg(fli1: EGFP) transgenic zebrafish. Zebrafish injected with PBS served as the negative control group. Zebrafish were maintained in 2 ml of 0.3× Danieau's solution (as previously described)^18^ in each well of 24‐well plates at 37℃. Living zebrafish were anesthetized with 0.04‐mg/ml tricaine and embedded in 3% methylcellulose. Serial images were captured using a Leica M205 FCA microscope (Leica Microsystems, Wetzlar, Germany). The metastatic patterns of CK‐MB‐1 cells throughout zebrafish were examined at ×8 magnification. The aforementioned zebrafish use protocol was reviewed and approved by the Institutional Animal Care and Use Committee of National Cheng Kung University (107281).

### 2.9 Xenograft heterotopic mouse tumor model

Two types of immunodeficient mice were chosen, namely, NOD.CB17‐*Prkdc^scid^*/JNarl (NOD SCID) and NOD. Cg‐*Prkdc^scid^Il2rg^tm1Wjl^*/YckNarl mice (Advanced Severe Immunodeficiency, ASID, National Laboratory Animal Center, Narlabs, Taiwan). CK‐MB‐1 cells (5 × 10^6^) in 100 μl of PBS were inoculated subcutaneously into the flank of each mouse at the age of 7 weeks in both the NOD SCID and ASID models. Tumor size was measured in length and width. Tumor volume was calculated using the equation (length × width^2^)/2. NOD SCID mice were randomly allocated to the control or trastuzumab group when tumors reached an average size of 150–200 mm^3^ (*n* = four per group). Mice in the control group received PBS intraperitoneally twice weekly, whereas mice in the trastuzumab group received intraperitoneal trastuzumab at a twice‐weekly dose of 30 mg/kg/d. Tumors were measured with calipers, and tumor volume was determined volume twice weekly. After 4 weeks of treatment, mice were sacrificed 24 h after the eighth administration. Tumors were harvested and prepared as formalin‐fixed/paraffin‐embedded tissues. The aforementioned mouse use protocol was reviewed and approved by the Institutional Animal Care and Use Committee of National Cheng Kung University (107033).

### 2.10 IHC staining

Paraffin sections were cut and mounted on silanized slides. Slides were melted at 65°C and dipped into xylene to remove the paraffin. After rehydrating tissues, slides were further dipped into a fresh aqueous solution of 3% peroxide in methanol. Heat retrieval was performed using citrate buffer in an autoclave for 10 minutes. The sections were then exposed to primary antibodies against ERα and HER2 diluted with Dako Antibody Diluent with Background Reducing Components (Agilent Technologies, Santa Clara, CA, USA). The signals were detected using Dako REAL™ EnVision™ Detection System (Agilent Technologies), and sections were subsequently counterstained with Mayer's hematoxylin and mounted (Malinol, Muto Pure Chemicals, Japan).

### 2.11 Statistical analysis

Experimental results assessing proliferation of the three cell lines under various treatment conditions are presented as bar charts. Each bar is the mean value of the data, and error bars represent the standard error of the mean (SEM). The growth curves of tumor xenografts present the tumor volume at check points of treatment. Each plot represents the mean tumor volume, whereas error bars represent the SEM. The final mean tumor volume in two groups was examined using the Mann–Whitney *U* test loaded in GraphPad Prism version 7.00 for Windows (GraphPad Software, La Jolla CA, USA, www.graphpad.com). *p* values less than 0.05 were considered statistically significant.

## 3 RESULTS

**FIGURE 1:**
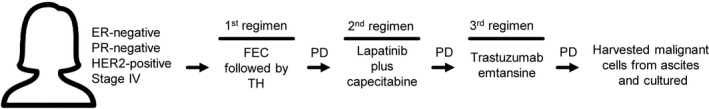
Summary of patient treatment. Before the development of malignant ascites, the patient received three lines of anti‐HER2 treatment for metastatic disease and following disease progression. FEC, fluorouracil, epirubicin, and cyclophosphamide; TH, docetaxel and trastuzumab; PD, progressive disease

**FIGURE 2:**
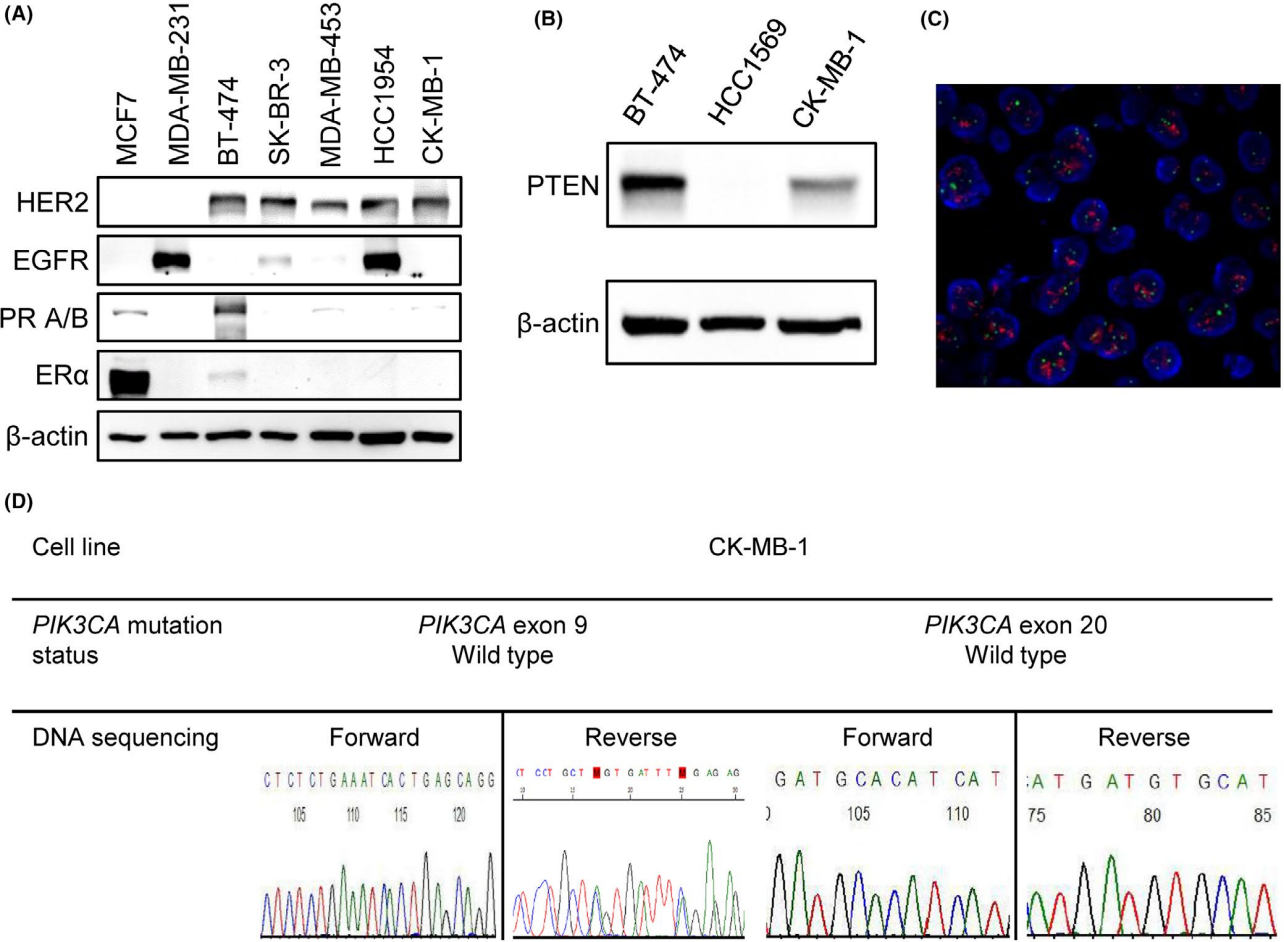
Characteristics of the CK‐MB‐1 cell line. (A) Western blotting confirmed HER2 positivity, ER/PR negativity, and no expression of EGFR in CK‐MB‐1 cells. (B) There was no loss of PTEN protein expression in CK‐MB‐1 cells. (C) HER2 gene amplification was detected by FISH. Orange signals were HER2 genes and green signals were chromosome 17 centromeres. (D) Sanger sequencing of CK‐MB‐1 cells did not reveal common *PIK3CA* mutation in exons 9 and 20. EGFR, epidermal growth factor receptor; ER, estrogen receptor; HER2, human epidermal growth factor receptor 2; PIK3CA, phosphoinositide‐3‐kinase, catalytic, alpha; PR, progesterone receptor; PTEN, phosphatase and tensin homolog

A 32‐year‐old female patient presented with hormone receptor‐negative/HER2‐positive metastatic breast cancer. She received an anthracycline‐based regimen followed by docetaxel plus trastuzumab as her first‐line treatment. She developed progressive disease during anti‐HER2 treatment. Lapatinib plus capecitabine served as the second‐line regimen, followed by trastuzumab emtansine as the third‐line regimen when she again experienced disease progression. Malignant ascites was the main problem even after treatment with trastuzumab emtansine. We harvested breast cancer cells from ascites after obtaining the consent of the patient and the approval of the institutional review board (Figure 1). The isolated breast cancer cell line, named CK‐MB‐1, could be continuously maintained, and it retained its proliferative characteristics after thawing from storage. Western blotting revealed that CK‐MB‐1 retained the ER/PR‐negative/HER2‐positive subtype, no expression of EGFR, and no loss of PTEN protein expression (Figure 2A,B). The amplification of HER2 gene was detected by fluorescence in situ hybridization (FISH) revealing a HER2 copy number of 19.45 and a HER2/CEP17 ratio of 5.22 (Figure 2C). The result has been interpreted and confirmed by pathologists. We evaluated the *PIK3CA* status of CK‐MB‐1 cells via Sanger sequencing because a proportion of trastuzumab‐resistant tumors arise from this mutation.^6^ The result revealed no common *PIK3CA* mutation in exons 9 and 20 (Figure 2D). In addition, NGS was applied to evaluate possible major oncogenic alterations in 44 genes in CK‐MB‐1 cells (Table S1). No common pathogenic mutation in *PIK3CA* or *PTEN* was found. Three candidate mutations, namely, *EGFR* p. Asn158 = (substitution ‐ coding silent), *EGFR* p. Gln787 = (substitution ‐ coding silent), and *TP53* p. Leu206Trp fs*41 (deletion ‐ frameshift), were identified as potential pathogenic mutations in a literature review.^19^, ^20^, ^21^, ^22^ Otherwise, no other known mutation causing resistance to anti‐HER2 therapy was revealed in CK‐MB‐1 cells.

**FIGURE 3:**
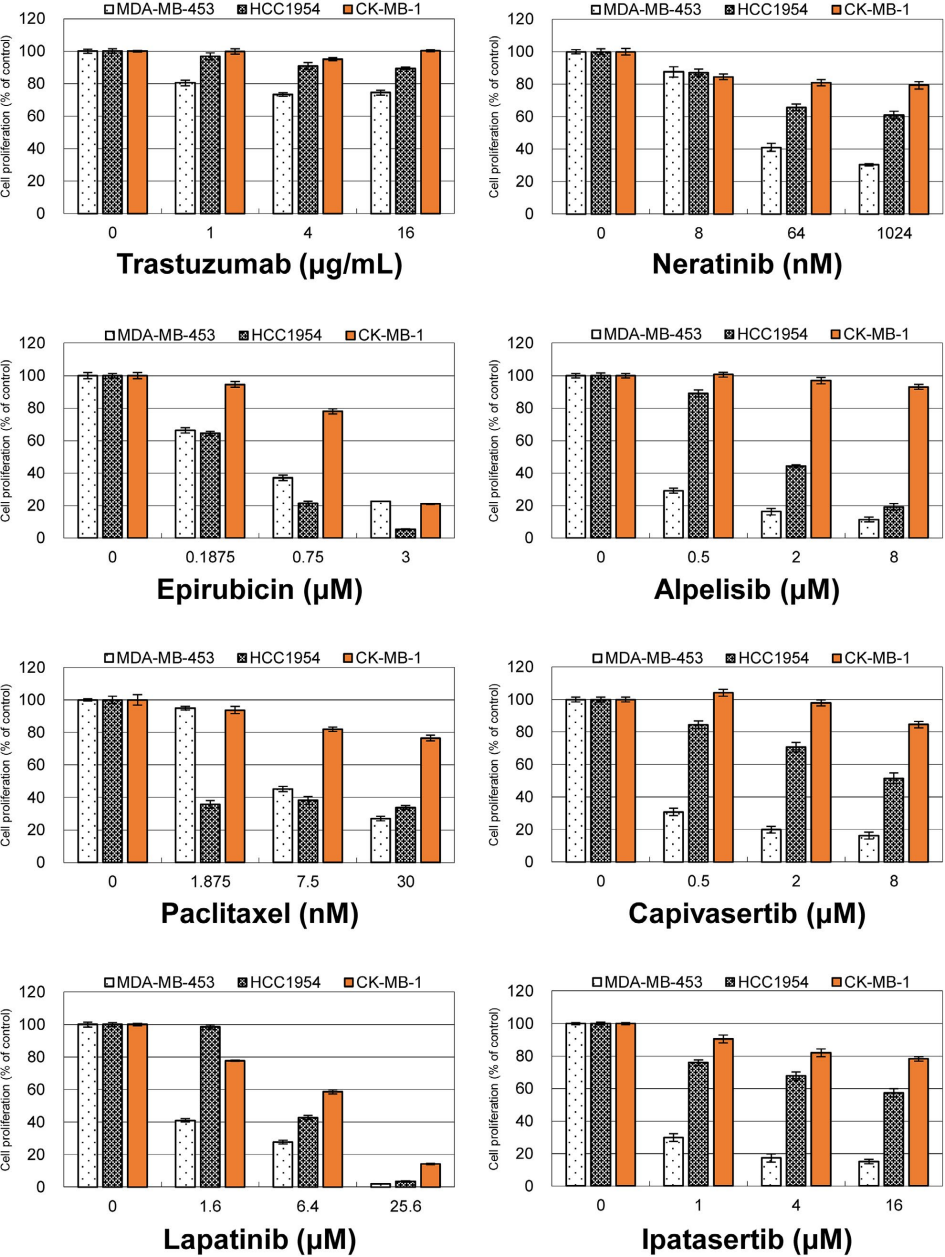
Antiproliferative effects of different drugs in three trastuzumab‐resistant cell lines. MDA‐MB‐453, HCC1954, and CK‐MB‐1 cells were treated with one anti‐HER2 monoclonal antibody, two chemotherapies, and five tyrosine kinase inhibitors targeting HER2, PI3K, or AKT. These drugs were less effective against the proliferation of CK‐MB‐1 cells than against the other two trastuzumab‐resistant cell lines. HER2, human epidermal growth factor receptor 2; PI3K, phosphoinositide‐3‐kinase

The drug‐resistant nature of CK‐MB‐1 cells was demonstrated via antiproliferative assays (Figure 3). We tested a series of drugs commonly used for HER2‐positive breast cancer treatment including one anti‐HER2 monoclonal antibody, two anti‐HER2 tyrosine kinase inhibitors, one PI3K inhibitor, two AKT inhibitors, and two chemotherapies for their antiproliferative effects in MDA‐MB‐453, HCC1954, and CK‐MB‐1 cells. In addition to its original resistance to anti‐HER2 treatments, CK‐MB‐1 cells exhibit relative resistance to chemotherapies drugs and even novel small‐molecule compounds compared with the findings in the two *PIK3CA*‐mutated cell lines. This result could also be validated by the results of other published cell line studies.^23^, ^24^

**FIGURE 4:**
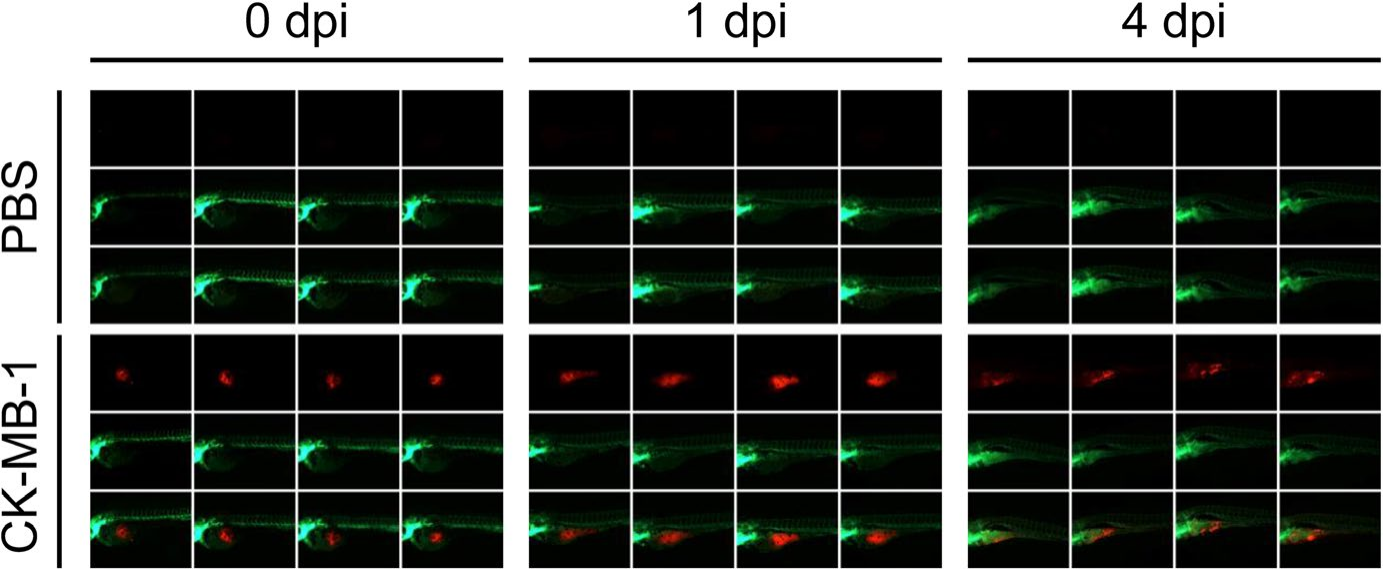
The zebrafish xenograft model of the CK‐MB‐1 cell line. Tg(fli1: EGFP) transgenic zebrafish were microinjected with PBS and CK‐MB‐1 cells (*n* = 4). Images of zebrafish were captured at 0, 1, and 4 dpi. CK‐MB‐1 cells were labeled with red fluorescence, in contrast to the green fluorescence from zebrafish. Migration and distant metastasis of CK‐MB‐1 cells to the tails of zebrafish could be observed at 4 dpi. There was no obvious deformity of zebrafish in the CK‐MB‐1 group in comparison with the findings in the PBS group. dpi: days post‐injury; PBS, phosphate‐buffered saline

To provide a comprehensive model for evaluating potential drugs for treating *PIK3CA*/*PTEN*–wild‐type, HER2‐positive, trastuzumab‐resistant breast cancer, we established xenograft tumors in zebrafish and immunodeficient mice. The temperature of the breeding system was higher than the usual setting to accelerate the growth rate of CK‐MB‐1 cells. Thus, the spreading pattern of cancer cells could be observed before zebrafish started to develop natural colors. Under such conditions, most zebrafish could survive until 4‐day post‐injury (dpi). The transgenic zebrafish emitted green fluorescent in their blood vessels, in contrast to the red fluorescence emitted by CK‐MB‐1 cells. The migration and spread of CK‐MB‐1 cells could be observed at 1 dpi (Figure 4). Metastases represented as red spots in the tails of zebrafish were vivid at 4 dpi (Figure S1). Compared to the findings in the PBS group, there was no obvious deformity of zebrafish injected with CK‐MB‐1 cells.

**FIGURE 5:**
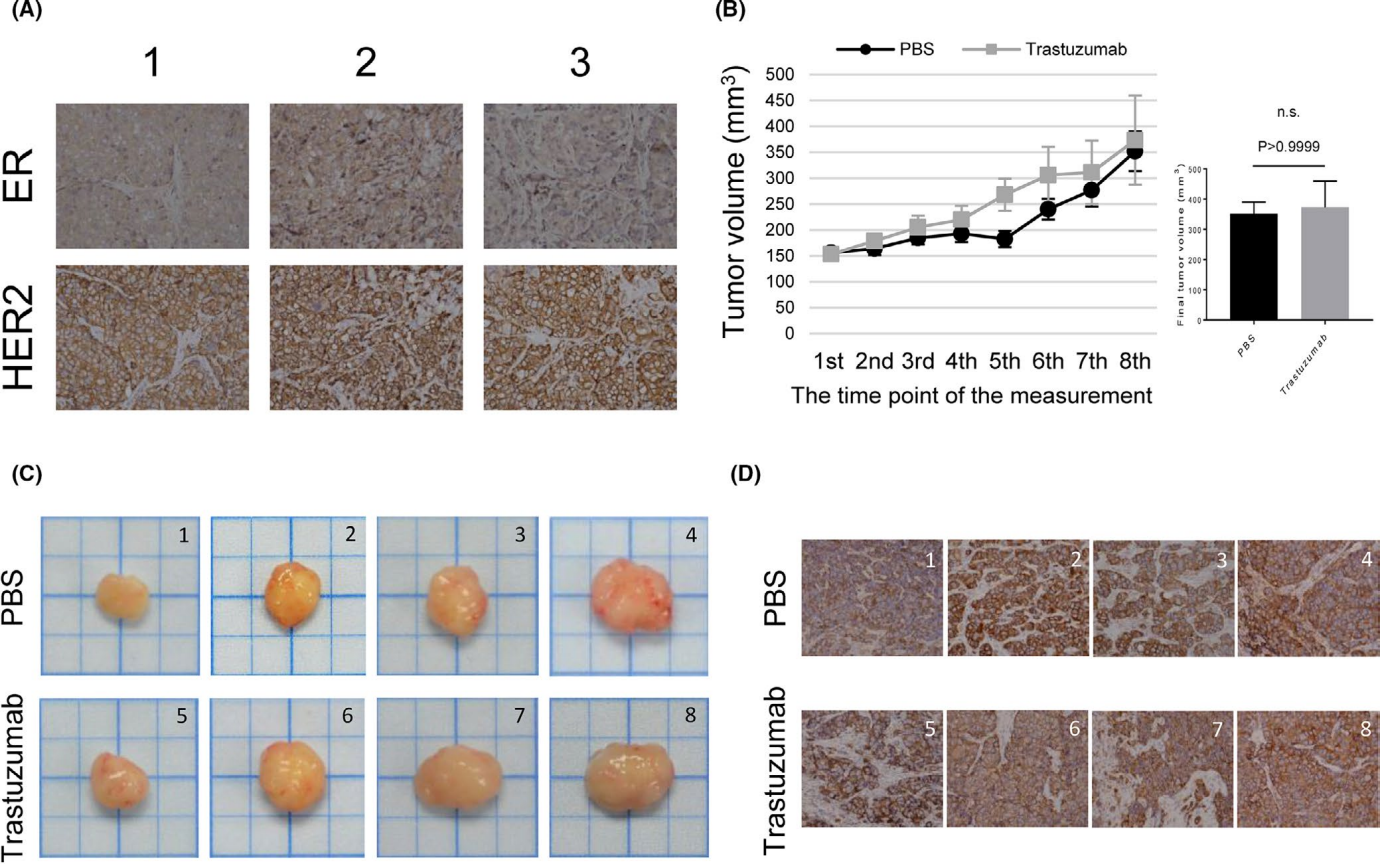
Xenograft mouse models of the CK‐MB‐1 cell line. (A) IHC staining revealed that CK‐MB‐1 xenografts maintained the same ER‐negative/HER2‐positive profile of the parental cell line in the ASID mouse model. (B) NOD‐SCID mice carrying CK‐MB‐1 xenografts were intraperitoneally injected with PBS or trastuzumab (*n* = four per group). The sizes of xenografts were recorded and plotted according to the protocol. Each plot was the mean of xenografts, and error bars represented the SEM. Xenografts were harvested on the next day after the eighth injection. The final tumor volume of two treatment groups was statistically analyzed using the Mann–Whitney *U* test. *P* values less than 0.05 were considered statistically significant. (C) Harvested xenografts were photographed and arranged in the order of final tumor size in each treatment group. (D) HER2 staining remained positive regardless of treatment in every xenograft harvested from NOD‐SCID mice. ER, estrogen receptor; HER2, human epidermal growth factor receptor 2; IHC, immunohistochemical; n.s., not significant; PBS, phosphate‐buffered saline; SEM, standard error of the mean

We also established heterotopic xenograft ASID and NOD SCID mouse tumor models using CK‐MB‐1 cells. The growth of xenografts was observed, and the size of xenografts reached 150 mm^3^ within 4–6 weeks. All xenografts were harvested and prepared for immunohistochemical (IHC) staining for ER and HER2. ER negativity and HER2 positivity were confirmed in vivo (Figure 5A). Meanwhile, tumor size was compared between the PBS and trastuzumab groups after 4 weeks, and no significant difference was observed (Figure 5B), illustrating that the CK‐MB‐1 xenografts were resistant to trastuzumab. Images of the excised tumors are shown in Figure 5C. NOD SCID mouse xenografts were also prepared for ER and HER2 staining. Despite treatment with trastuzumab, HER2 overexpression and ER negativity remained (Figure 5D and Figure S2).

## 4 DISCUSSION

The survival of patients with HER2‐positive metastatic breast cancer has markedly improved since the introduction of anti‐HER2 therapies. However, disease progression occurs in most patients receiving the standard first‐line treatment.^4^ PI3K and PTEN mutation contributes in part to the mechanism of resistance.^25^ Most trastuzumab‐resistant cell lines available from biobanks belong to this category. The majority of patients with progressive disease after anti‐HER2 treatment lack a druggable mutation. Thus, HER2‐targeting strategies serve as the foundation of treatment in the second and later lines.^26^, ^27^, ^28^ Therefore, broadening the variety of trastuzumab‐resistant cells is important for the development of future drugs.

We confirmed that CK‐MB‐1 cells are ER‐negative and HER2‐positive without PTEN loss or *PTEN*/*PIK3CA* mutation. Although we identified two potential pathogenic *EGFR* mutations in CK‐MB‐1 cells via NGS, the antiproliferative effects of lapatinib or neratinib were not promising. These two drugs inhibit the function of EGFR and HER2. However, there was no report regarding to the efficacy of lapatinib and neratinib in the two *EGFR* mutations of CK‐MB‐1 cells. Therefore, the two *EGFR* mutations did not sensitize CK‐MB‐1 cells to EGFR tyrosine kinase inhibitors. Since the EGFR mutations are synonymous, they are unlikely to affect protein function and behavior of the cell line. Moreover, the poor responses of CK‐MB‐1 cells to alpelisib, capivasertib, and ipatasertib suggested that the major survival mechanism of this cell line is independent of the PI3K/AKT pathway.

Establishing animal models with cell lines is crucial for testing the treatment effects of drugs in vivo. The CK‐MB‐1 zebrafish model provides researchers a tool for examining interventions to block metastasis. CK‐MB‐1 xenograft models in ASID or NOD SCID mice could offer more routes for administering drugs and additional strategies for evaluating the response of tumors. This ineffective treatment suggested the antibody‐depended cell cytotoxicity related to trastuzumab as previously reported being not observed in the CK‐MB‐1 mouse model.^29^ This implicates that CK‐MB‐1 cell‐based models can be used to test the efficacy of anti‐HER2 antibody‐drug conjugates and other novel drugs.

In summary, CK‐MB‐1 cells represent a stable trastuzumab‐resistant breast cancer cell line that is suitable for in vitro and in vivo experiments. The resistance of this cell line to anti‐HER2 drugs does not depend on the HER2/PI3K/PTEN/AKT pathway. Thus, these cells will permit a comprehensive approach to evaluating potential drugs targeting *PIK3CA*/*PTEN*–wild‐type, HER2‐positive, trastuzumab‐resistant breast cancer, which comprises a large proportion of the trastuzumab‐resistant breast cancer population. We believe that it will be beneficial to incorporate CK‐MB‐1 cells into the development of novel HER2‐targeted therapies in the future.

## CONFLICT OF INTEREST

The authors declare that they have no conflict of interest.

## AUTHOR CONTRIBUTIONS

WPC planned the research, performed and analyzed the experiments, and wrote the manuscript. WLH planned, performed, and analyzed some parts of the experiments and wrote certain parts of the manuscript. WAL analyzed some parts of the experiments. WLH performed some parts of the experiments. YYL performed some parts of the experiments. WCS planned the experiments and supervised the project.

## Supporting information

Fig S1

Fig S2

Table S1

## Data Availability

Data sharing not applicable, and no new data generated.
