# Supplementary figures and images for: Development of the CK‐MB‐1 trastuzumab‐resistant HER2‐positive breast cancer cell line and xenograft animal models
Source: Cancer Med. 2021 Mar 5;10(7):2370–9. doi: 10.1002/cam4.3824 (PMC7982635; doi:10.1002/cam4.3824)

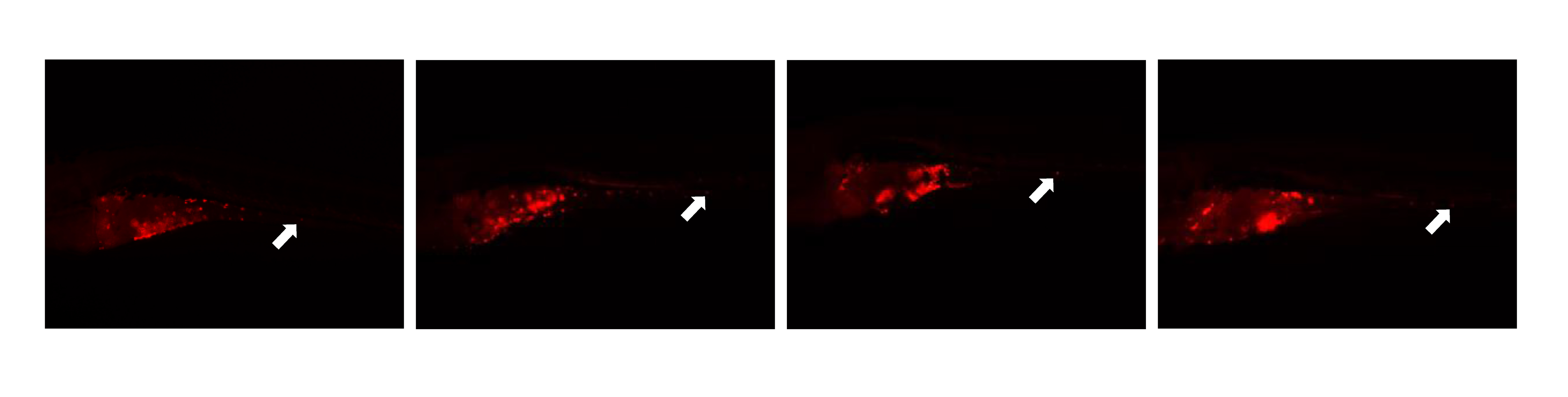

Supplement: Supplementary file 1 — Fig S1 [file CAM4-10-2370-s003.jpg]

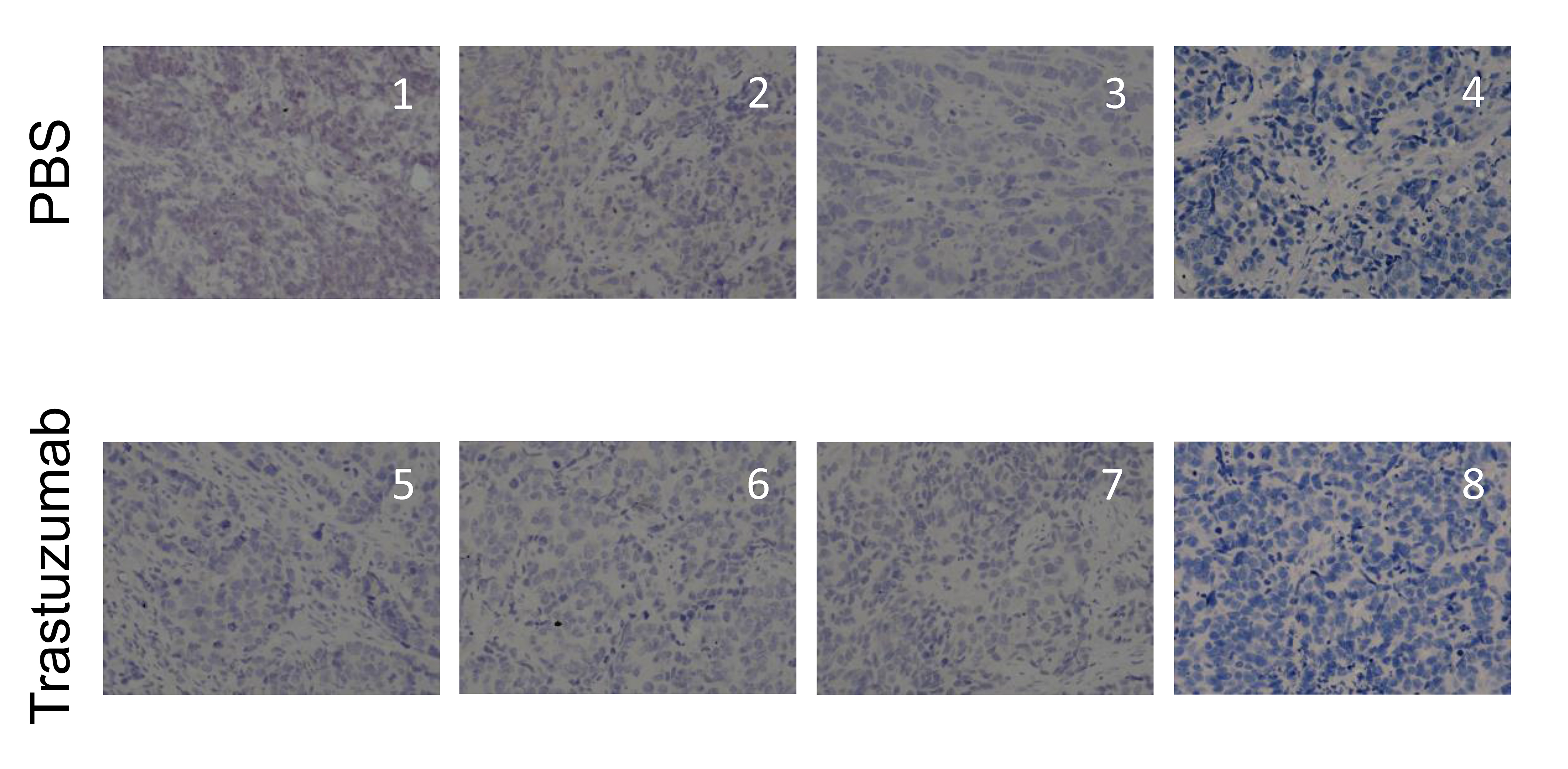

Supplement: Supplementary file 2 — Fig S2 [file CAM4-10-2370-s002.jpg]
