# Supplementary material for: Development of the CK‐MB‐1 trastuzumab‐resistant HER2‐positive breast cancer cell line and xenograft animal models
Source: Cancer Med. 2021 Mar 5;10(7):2370–9. doi: 10.1002/cam4.3824 (PMC7982635; doi:10.1002/cam4.3824)
Supplement: Supplementary file 3 — Table S1 [file CAM4-10-2370-s001.docx]

Supporting Table 1: Summary of NGS results analyzed with Human Breast Cancer GeneRead DNAseq Targeted Panel V2

| Genes | 44 |
| --- | --- |
| Amplicons | 2915 |
| Total reads | 1860898 |
| Median read depth in target region | 315 |
| Mean read depth in target region | 659 |
| % of bases covered at >= 30x | 88 |
| % of bases covered at >= 100x | 75 |
| % of bases callable according to GATK CallableLoci Walker | 91 |
| SNPs / MNPs | 387 |
| Insertions / Deletions | 34 |
| High confidence variants (variants that pass all filters) | 287 |
